# A novel approach to emotion recognition using local subset feature selection and modified Dempster-Shafer theory

**DOI:** 10.1186/s12993-018-0149-4

**Published:** 2018-10-31

**Authors:** Morteza Zangeneh Soroush, Keivan Maghooli, Seyed Kamaledin Setarehdan, Ali Motie Nasrabadi

**Affiliations:** 1 Department of Biomedical Engineering, Science and Research Branch, Islamic Azad University, Tehran, Iran; 2 Control and Intelligent Processing Centre of Excellence, School of Electrical and Computer Engineering, College of Engineering, University of Tehran, Tehran, Iran; 3 Department of Biomedical Engineering, Faculty of Engineering, Shahed University, Tehran, Iran

**Keywords:** Emotion identification, Local subset feature selection, Machine learning methods, Independent component analysis, Dempster Shafer theory, Brain computer interactions

## Abstract

**Background:**

Emotion recognition is an increasingly important field of research in brain computer interactions.

**Introduction:**

With the advance of technology, automatic emotion recognition systems no longer seem far-fetched. Be that as it may, detecting neural correlates of emotion has remained a substantial bottleneck. Settling this issue will be a breakthrough of significance in the literature.

**Methods:**

The current study aims to identify the correlations between different emotions and brain regions with the help of suitable electrodes. Initially, independent component analysis algorithm is employed to remove artifacts and extract the independent components. The informative channels are then selected based on the thresholded average activity value for obtained components. Afterwards, effective features are extracted from selected channels common between all emotion classes. Features are reduced using the local subset feature selection method and then fed to a new classification model using modified Dempster-Shafer theory of evidence.

**Results:**

The presented method is employed to DEAP dataset and the results are compared to those of previous studies, which highlights the significant ability of this method to recognize emotions through electroencephalography, by the accuracy of about 91%. Finally, the obtained results are discussed and new aspects are introduced.

**Conclusions:**

The present study addresses the long-standing challenge of finding neural correlates between human emotions and the activated brain regions. Also, we managed to solve uncertainty problem in emotion classification which is one of the most challenging issues in this field. The proposed method could be employed in other practical applications in future.

## Introduction

A fundamental controversy that has been driving extensive research in phycology and neuroscience today concerns what emotion really is. Though seemingly simple, the definition of emotion has in fact remained as an area of little consensus. Most often, the term emotion refers to a psycho-physiological process triggered by conscious and unconscious perception of an object or situation and is commonly associated with mood, temperament, personality, disposition, and motivation. Emotion is central to almost any interpersonal communication and is generally expressed through both verbal and nonverbal cues. Quite undeniably, emotions pervade every aspect of human life, having profound influences on our actions as well as our perceptions. This has led to the development of systems that attempt to recognize and interpret human affects to establish affective human–computer interactions (HCI). However, as yet most human–computer interaction systems are far from being emotionally intelligent and thus, tend to fail to distinguish and discriminate emotional states and decide upon following proper actions. Therefore, affective computing, as a growing field, sets its goal to bridge this gap by identifying emotional states using the exhibited cues and generating proper responses [1].

Over the past few years, the studies on emotion recognition through EEG have received increasing attention and are now extending into interdisciplinary fields that range from psychology to different branches of engineering. They typically include preliminary researches on emotion theories and applications to affective BCIs [2, 3], which allow for identifying, analyzing and responding to user’s affective states based on physiological signals.

Emotion recognition is a key step towards emotional intelligence in advanced human–machine interaction. It is mainly served through analyzing either emotional expressions or physiological signals. The former refers to any observable emotional cues that communicates emotion, while the latter, which has so far received little attention, includes information that lies in signals originating from the central and peripheral nervous system such as blood pressure, respiration, skin conductivity, pupil dilation, heart rate, and so forth. In the field of affective computing, different signals have been drawn into focus to study emotion recognition. For a comprehensive review of emotion recognition methods, one can refer to Calvo and D’Mello [4].

EEG is largely employed to investigate the brain activity associated to emotion since it allows for the identification of immediate responses to emotional stimuli and could potentially reflect emotional states in a relatively cost-and computation-effective manner. Nevertheless, emotion recognition based on EEG could come across as challenging, factoring in the fuzzy boundaries and individual differences related to emotions. Furthermore, it seems theoretically unlikely to obtain the correct category for an EEG that corresponds to different emotional states since emotion is generally regarded as a function of various variables such as time, culture, and race [5].

With the rapid growth of micro-nano technologies and embedded systems, it is no longer far-fetched to have BCI systems ported from a laboratory demonstration to real-life applications. Thanks to new advances in materials and integrated electronic systems technologies, a new generation of dry electrodes and embedded systems have been developed to fulfill the basic needs for increased practicability, wearability, and portability of BCI systems in real-world environments [6, 7].

Recently, an increasing number of affective computing researches have been conducted with the aim of building computational models that employ EEG features to estimate emotional states. A review of such models can be found in [8], the work of Kim et al. Affective neuroscience seeks, among other goals, to study the neural associations between human emotions and the obtained brain activity, particularly such EEG signatures of emotion that are more likely to be shared across individuals. Researches in the literature suggest that while processing modules for particular emotions appear to be non-existent, finding neural signatures of emotions, signified by a distributed pattern of brain activity [9], seem theoretically and practically possible. Mauss and Robinson [10] came to the conclusion that the emotional state tends to involve circuits as opposed to any isolated brain region. Furthermore, it is widely believed that identifying neural patterns which are common across individuals and are also stable across sessions can contribute significantly to EEG-based emotion recognition. On the other hand, cortical activity following emotional cues is attributed to the lateralization effect. Schmidt and Trainor [11] discovered that valence and intensity could be identified by the pattern of asymmetrical frontal EEG activity and the overall frontal EEG activity, respectively. Muller et al. noticed a correlation between gamma power and a negative valence over the left temporal region [12]. Bringing into attention the relation between frontal EEG asymmetry and approach and withdrawal emotions, Davidson and Fox [13] and Davidson [14] demonstrated that the left frontal activity mirrors heightened approach tendencies, while withdrawal tendencies are reflected in the right frontal activity. Nie et al. in [15] noted the prevalence of the subject-independent features attributed to positive and negative emotions in the right occipital lobe and parietal lobe for the alpha band, the central site for the beta band, and the left frontal lobe and right temporal lobe for the gamma band. Balconi et al. suggested that valence and arousal rating affect frequency band modulations such that high arousal and negative or positive stimuli can trigger an increased response [16].

Despite all earlier efforts, the lack of recognizable neural signatures of emotion has continued to be a major barrier. Finding such a strategy to settle this issue will be a breakthrough of substantive significance, paving the way for several subsequent developments in psychology, cognitive sciences, and other relevant fields. Therefore, the current study, through combining novel approaches and proposing a new structure, aims to identify the active regions using suitable electrodes with acceptable level of accuracy.

According to the Circumplex Model, emotions are distributed in a two-dimensional circular space where the vertical and horizontal axes represent arousal and valence dimensions respectively. The two axes intersect at one point, dividing the space into four main quadrants which are used in labeling data in this research. The current study aims to manage the novel methods, and propose a structure for active neural structures associated with specific emotions and to present an optimal strategy for applying these approaches to achieve an accurate classification of emotions. The proposed structure makes use of novel and optimized algorithms for extracting emotions in an effective and organized manner to bring about the best possible results. Using EEG channels, this study attempts to identify brain regions that are active when experiencing a specific emotion. To this end, ICA algorithm is employed to remove artifacts and extract the independent components. Then, based on the extracted mapping, channels will receive a corresponding value.

**Fig. 1:**
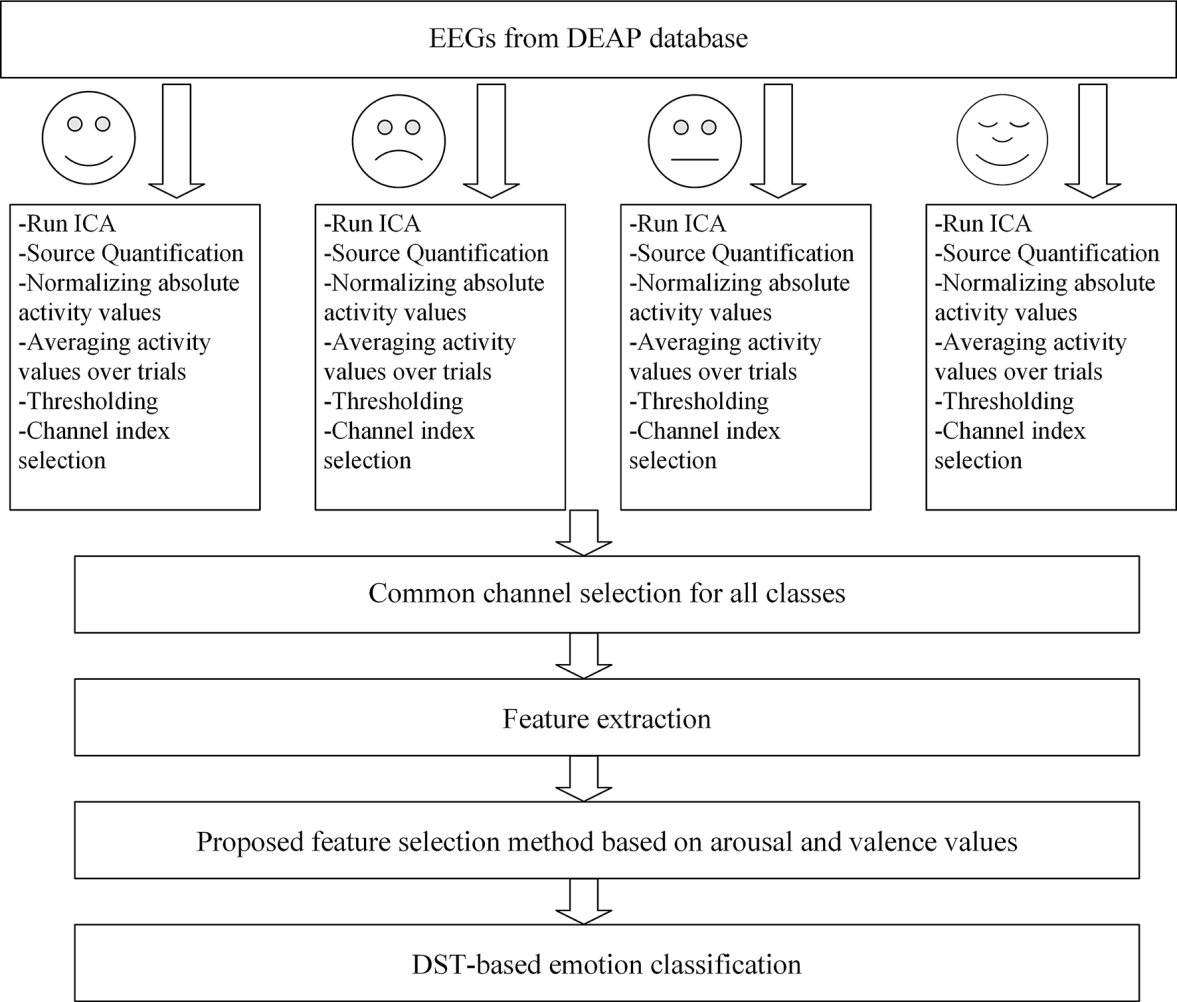
Block diagram of the proposed approach for emotion detection

In this setting, the absolute values of active and inactive regions will be obtained. The normalized value as well as the average value of each channel are then calculated and compared to a threshold value, which leads to the selection of active channels that are suited to our task. The process is repeated for each of the four classes of emotions, choosing the informative channels. The channels that are common in all classes would further be selected to allow for extraction of the proposed features. The proposed method would proceed to implement the feature selection along the arousal and valence dimensions. In the end, emotions are classified using the optimized Dempster Shafer method. Figure 1 illustrates the block diagram of the presents approach.

## Materials and methods

### Dataset used

The database contains all recorded signal data, frontal face video for a group of the participants, subjective ratings from the participants as well as the subjective ratings from the initial online subjective annotation and the list of 120 videos used. Koelstra et al. built the DEAP database aiming at examining spontaneous human affective states that are specifically induced by music videos [1]. The dataset contains 32 healthy participants half males and half females, with the age range of 19 to 37 (mean = 26.9). For each participant, 40 videos were separately presented in 40 trials with the EEG and peripheral physiological signals simultaneously recorded. In each of them, the index of the current trial was first displayed for 2-s; and a consecutive 5-s recording proceeded as the baseline condition; then the music video was shown for 1 min; finally, the subjective-ratings on arousal, valence, liking and dominance scales were collected.

### Channel selection

**Fig. 2:**
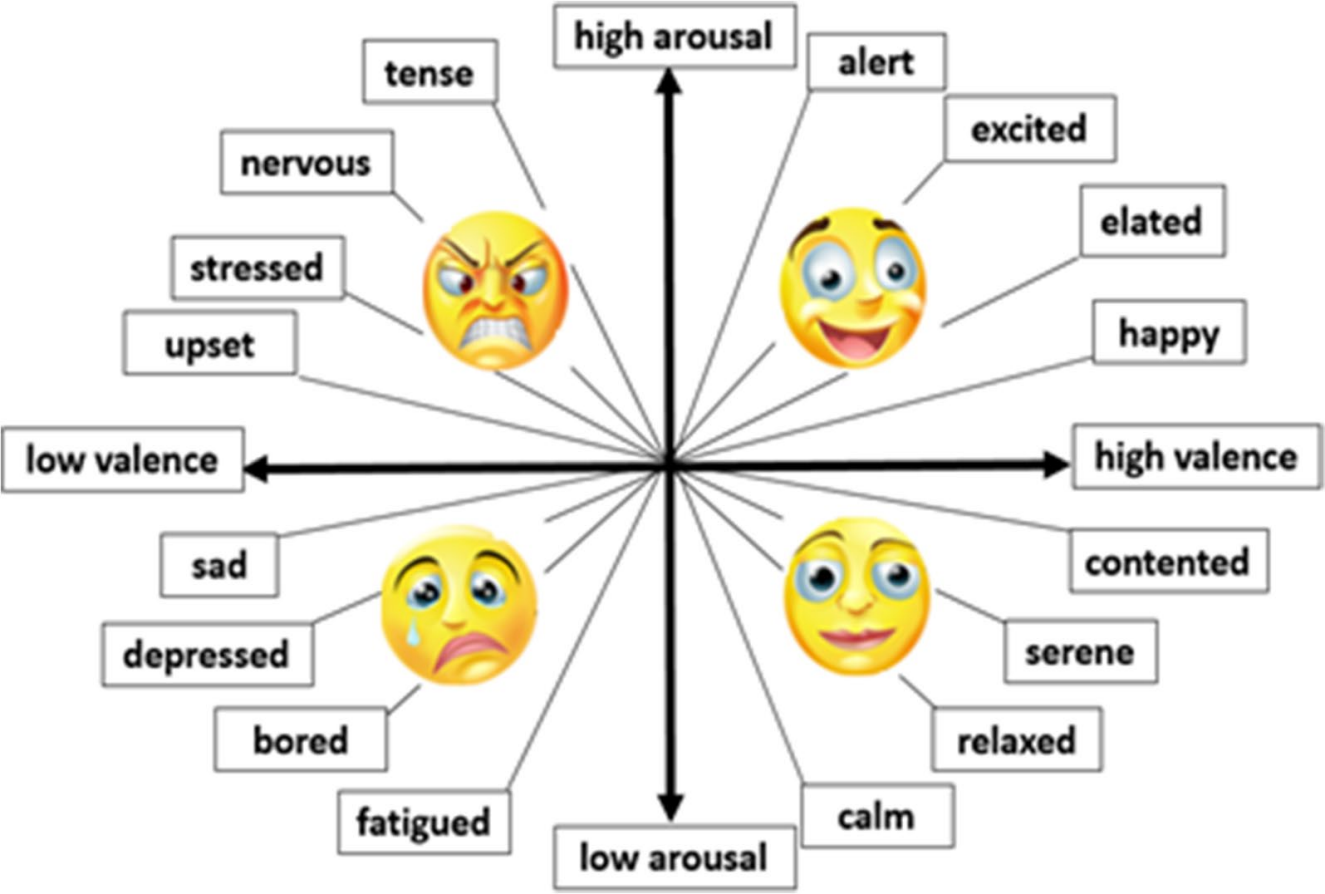
Arousal-valence plane and label distribution for DEAP dataset

In this section, we propose a method to select most active channels associated with different states of emotions. As mentioned before, emotions can be described through the arousal-valence plane which allows considering four different regions as emotional states. Figure 2 shows the arousal-valence plane as well as the emotional states. Here, we have simply named these four quarters as: Quarter 1 (Q1), Quarter 2 (Q2), Quarter 3 (Q3) and Quarter 4 (Q4).

Arousal and valence distribution in DEAP dataset is also represented in such plane. 1280 samples (32 individuals, each 40 trials) are almost uniformly distributed in arousal-valence plane indicating that there are adequate numbers of samples in each class. This section aims to determine neural correlates between each emotional state, i.e. class, and the registered EEG signals and thus selecting the EEG channels that display appreciable higher activity.

EEG activity can be demonstrated using blind source separation (BSS) methods like ICA. The current study applies Runica as well as second order blind identification (SOBI), JADE and COMBI which are believed to be the best BSS methods for EEG signal processing applications in several surveys such as [17, 18]. EEGs for each class are first fed into BSS methods to get sources separated. 32 EEG sources (i.e. independent components) are estimated and reconstructed in each BSS method. Based on the surveys, we employed the mentioned BSS methods to evaluate and compare them in terms of emotion recognition and emotion-related neural activity.

**Fig. 3:**
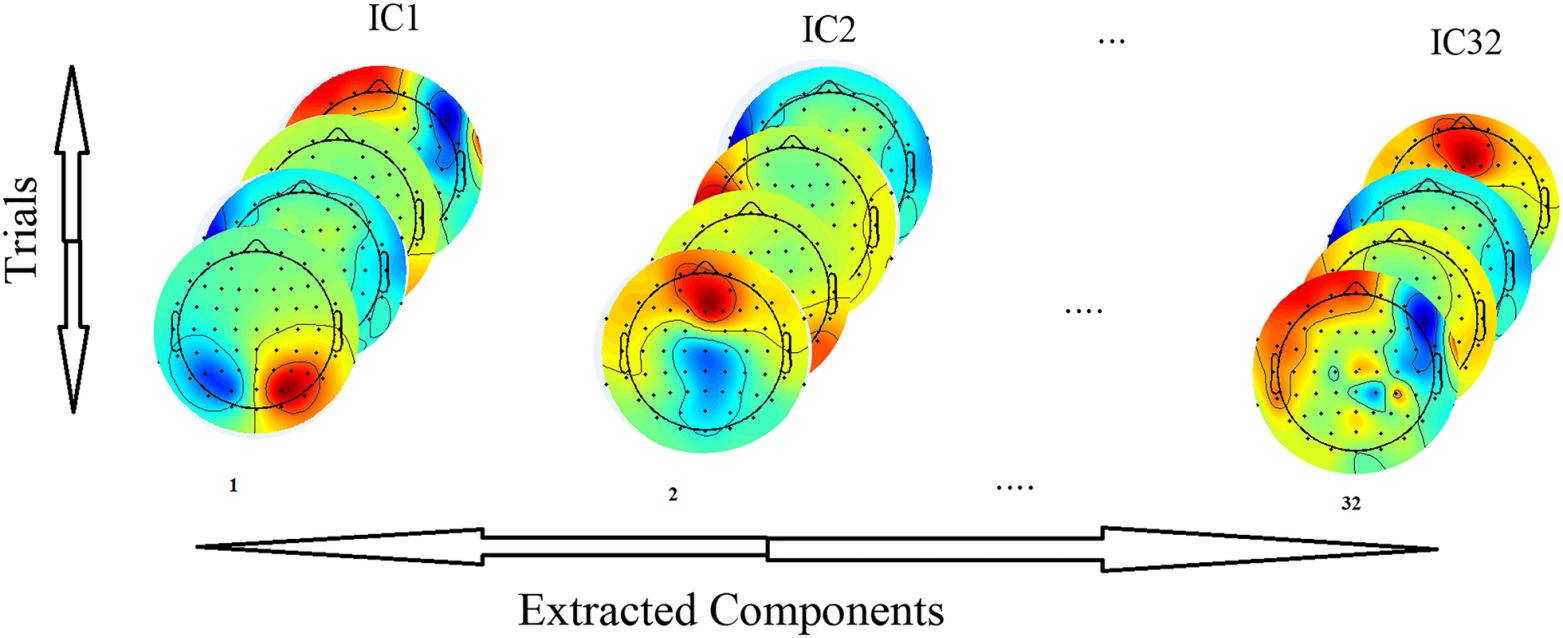
Average score of each ICA component for all of trials

**Fig. 4:**
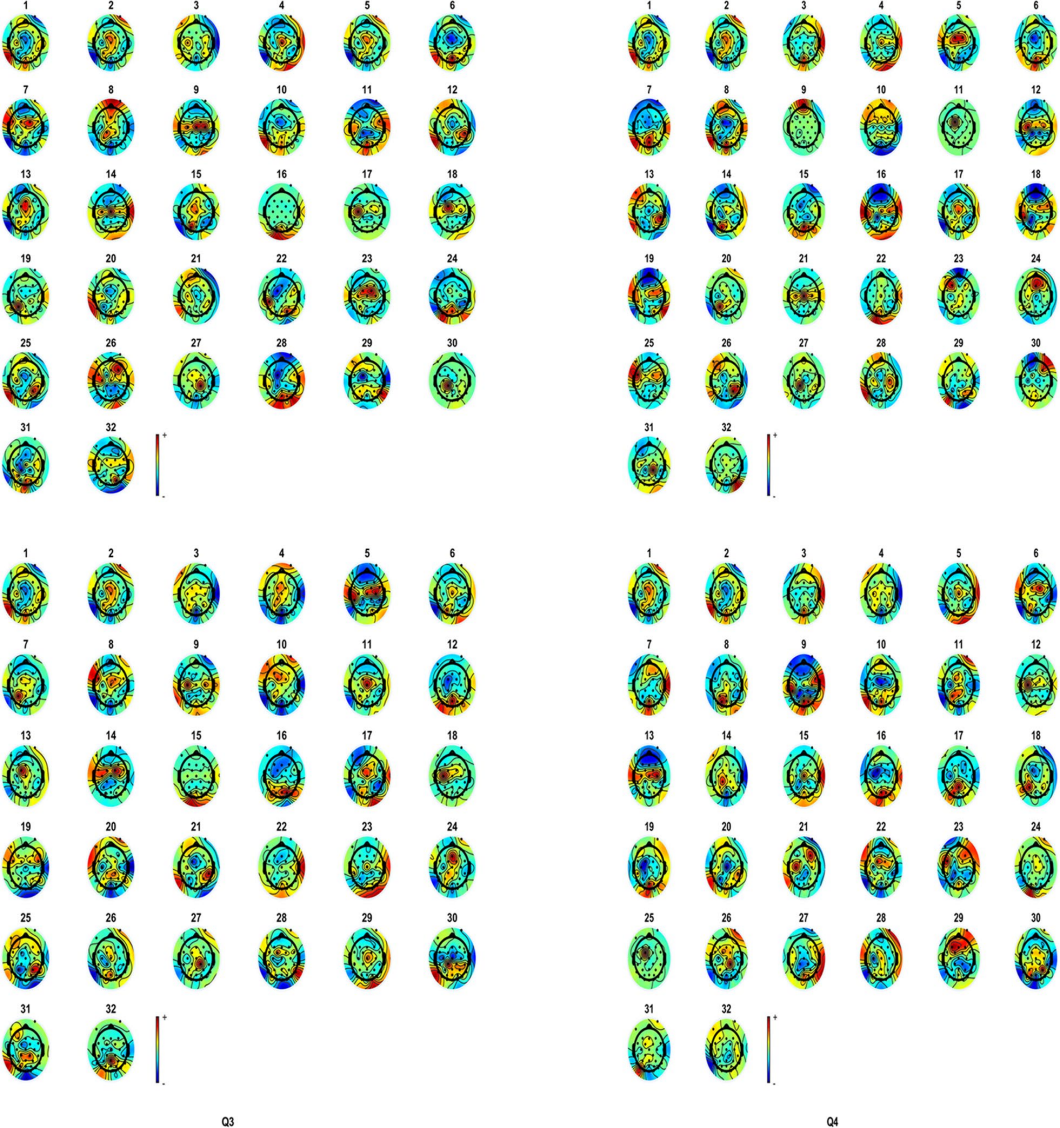
Average score of 32 components of ICA for: **a** Q1, **b** Q2, **c** Q3, **d** Q4

It should be noted that, EEGs in DEAP database have been preprocessed before and it has been observed that no noticeable artifacts or noises exist which means all extracted sources are correspondent to neural activity. Neural activity is estimated for each component and then averaged over samples in each emotional class to have the average activity maps for each emotional state. Figures 3, 4 depict the average activity mapping for 32 channels in Q1–Q4 emotional states, respectively.

EEG source separation and topographic mapping are carried out using EEGLAB in this study. Activity values are then normalized with respect to minimum and maximum activity in the dataset. All normalized activity values vary in the range of [− 1, 1]. Since both active and inactive regions (i.e. channels) are of importance, we focus on the absolute value of neural activity to find most active and significant channels in emotional changes.

Channels with activity values being higher than a specific threshold are considered as “Emotional Channels” in each class. This threshold is determined based on a trial and error procedure to achieve best classification performance.

Since only the selected channels are taken to the next step, this method holds promise to be less time-consuming and more accurate. Channel indices for each emotional state are determined with respect to channel activity and only common channels among all four classes are considered to be processed in the rest of the proposed method.

### Feature extraction

Prior to classification of samples, effective features are extracted from selected channels. Several studies like [19–34] have applied different features while working on DEAP database. This study makes use of features that have been previously proposed as well as nonlinear features which are believed to be effective in emotion recognition. These features are estimated from the entire 1-min selected EEG channels which were explained in the previous section. Since we concentrate on nonlinear features which are mostly extracted from the signal phase space, we need longer windows (e.g. 1 or 2 min at sampling frequency of 256 or 128 Hz) to reconstruct the EEG phase space. Moreover, some features such as different kinds of entropies need at least 4000 samples to be estimated correctly and precisely.

Table 1 lists the proposed features and the abbreviations.

**Table 1:** Most common features in emotion recognition through EEG

#	Feature description	Abbreviation	Explained in
1	Correlation dimension	CD	[22, 37, 38]
2	Fractal dimension	FD	[40, 41, 49]
3	Largest Lyapunov exponent	LLE	[37, 40, 41]
4	Sample entropy	SpEn	[33, 36]
5	Recurrence rate	RR	[35, 39]
6	Determinism	DET	[35, 39]
7	Average diagonal line length	L	[35, 39]
8	Entropy	ENT	[35, 39]
9	Differential entropy	DeEn	[19, 27]

For reasons of space, we avoid explaining these well-known features here. For more information, refer to the mentioned references.

### Local subset feature selection

**Fig. 5:**
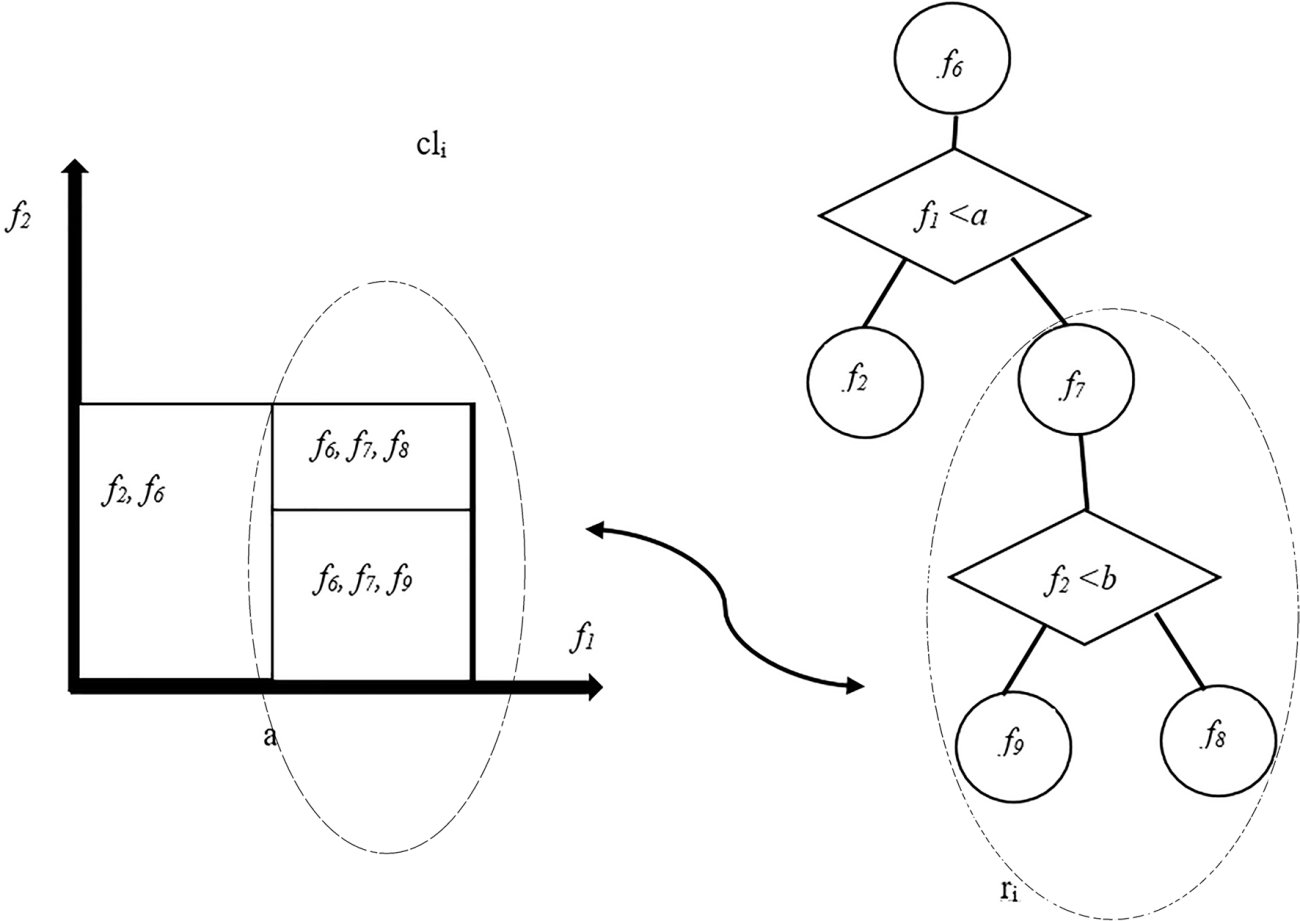
An instance from different localities and features in tree representation. The sub-tree *r*_*i*_ corresponds to the compound locality *cl*_*i*_ consisting of two single localities [42]

This section focuses on feature selection algorithm. Taking a close look at labels in this dataset, i.e. arousal and valence, we can select a number of informative features simply by considering these values. To this end, the current study benefits form one of the recent and successful feature selection methods called Bandit [42–47] where features are selected based on defined regions in the feature space. Turning the problem of feature selection into a sequential decision-making problem, this method applies the concept of feature tree, as a developed model of decision trees, to divide the sample space into a few localities and assign features to each of them. In addition to splitting and leaf nodes in a typical decision tree, a feature tree includes another type of node named ‘feature node’, which shows a feature that is attributed to all of its decedents and can have no more than one child. A Compound Locality further refers to a sub-tree corresponding to a set of neighbor localities. This representation simplifies the selection of similar features since neighbor localities are more likely to share mutual features, which will be factored together in the parent feature node. Figure 5 depicts a sample feature tree where the feature nodes are represented by a circle with a single feature inside, a splitting node by a rectangle containing a feature and a threshold, and localities by leaves. In order for the localities to be dependent on a limited number of features, it has been assuming that partitioning can be represented using a univariate binary decision tree [42].

Feature trees assign a sample, either training or test, to a descendant in the root repeatedly, based on the value of the corresponding feature, until it is assigned to a unique leaf. Accordingly, a subset of training samples and a subset of features, that is the set of feature nodes from the leaf to the root, are accumulated in each locality as the process precedes. For a test sample classification, it is first assigned to a locality according to the feature tree and is then classified in the locality through the corresponding features and training samples. To ensure an effective local feature selection, we employ a criterion which helps us compare different feature trees. We expect that the sample of different classes be separable in the new space formed by the selected features. With that in mind, *S* and *ft* are assumed to be the training set and the feature tree, respectively. Given *ft* and a random sample *x*, we can find the subset of *S* that belongs to the same locality as *x*. Let *s* *⊂ L*(*x, s, ft, k*) be the k-nearest neighbors of x among the members of this subset. The score of *ft* with respect to the training set *S* is computed as:

1 $$SCORE(ft) = \frac{1}{{\text{K} \cdot \left| \text{S} \right|}}\sum\limits_{{\text{x} \in \text{s}}} {} \sum\limits_{{\text{y} \in \text{L(x,s,ft,k)}}} {\left\{ \begin{aligned} & 1\;\text{label}\;(\text{y}) = \text{label}\;(\text{x}) \hfill \\ &0\;\text{otherwise} \hfill \\ \end{aligned} \right.}$$

where label (·) signifies the class of a sample.

In another perspective, each node of a feature tree is regarded as an equivalent of a state in the Reinforcement Learning (RL) machine, consisting of a sequence of nodes from the root to the current node. The RL agent selects an action for each state, which in this setting, means choosing the node type and the corresponding feature index. Accordingly, the set of all possible actions in each state is ${\text{Actions }} = \, \left\{ {f1, \, f2, \, .., \, Ff, \, S1, \, S2, \, \ldots , \, SF, \, T} \right\}$ with F being the number of features, *fi* and *si* showing a feature node and a splitting node respectively, and T being the terminating action, which finishes feature selection in the current node, leaving it as a leaf [42].

### Classification

Since emotions are described by arousal and valence values, we can consider four emotional quarters, i.e. Q1–Q4 classes, in two independent binary classifications. Q1, denotes samples with high valence-high arousal (HVHA). Similarly, Q2, Q3 and Q4 classes mark samples with low valence-high arousal (LVHA), low valence-low arousal (LVLA) and high valence-low arousal (HVLA), respectively. In this paper, we classify samples based on two feature subsets by two separate and independent multi-layer perceptron (MLP) neural networks. MLP is among the most popular classifiers in pattern recognition problems. This classification model works in two main steps: training and testing. In the training phase, weights are adjusted to achieve the least training error. Then, the test samples will be made use of to evaluate the classifier in the testing phase. Numerous studies have employed MLP to identify emotions [48–54].

As the feature selection procedure returns two different subsets as the output, we propose to employ Dempster-Shafer theory (DST) of evidence to combine two MLPs trained by two different feature subsets. DST is reported to be one of the most commonly used methods to reduce uncertainty and increase classification accuracy [38].

Introduced by Dempster and then modified by Shafer, DST is a widely used, theoretical framework which offers a way to handle imprecise, uncertain and partial information. In addition, this theory is applied to fuse different information sources and feature subsets [55]. Therefore, fusion of classifiers can also be performed with the help of this framework. Posterior probability values can be combined using DST and final decision could be made. This theory can reduce uncertainty and incompleteness and lead to a higher accuracy of classification by applying a combination rule for belief functions (Bel) of different information sources. These sources could be some experts or classification models trained by subsets of features. Different classifiers can be combined through this theory. Combination of classification models yields considerably better classification results. DST is explained as follows.

Let us suppose $\varphi = \{ s_{1} , s_{1} , \ldots , s_{m} \}$. The number of all possible subsets or hypothesis is $2^{\varphi } = \{ s_{1} ,s_{2} , \left\{ {s_{1} ,s_{2} } \right\}, \ldots , \left\{ {s_{1} ,s_{2} , \ldots , s_{m} } \right\}\}$. Bels (or mass values) could be defined for each subset. A mass value determines the degree of belief which is assigned to a specific subset. A Bel should satisfy the following conditions:

2 $$m\left( \phi \right) = 0$$

3 $$m\left( S \right) \ge 0, \quad \forall_{S \subseteq \varphi }$$

4 $$\mathop \sum \limits_{S \subseteq \varphi } m\left( S \right) = 1$$

With some assumption, we can consider posterior probabilities of classifiers as mass values. As it is mentioned, mass values have some characteristics. There are some methods to transfer the output of a classifier into mass functions [29]. In the current study, we have used softmax operator [38] which is defined as following:

5 $$m_{i} \left( {\left\{ {s_{j} } \right\}} \right) = \frac{{exp(R_{ji} )}}{{\mathop \sum \nolimits_{j = 1}^{C} R_{ji} }} \quad \, j = 1, \ldots ,C$$

In which $R_{ji}$ is the $j$th posterior probability value of $i$ th classifier. $C$ signifies the number of classes and $m$ indicates the mass value. Also the combination of mass values assigned by $n$ different independent sources can be performed through Dempster’s combination rule as follows:

6 $$m\left( S \right) = \frac{{\mathop \sum \nolimits_{{S_{1} \mathop \cap \nolimits \ldots \mathop \cap \nolimits S_{n} = S}} \mathop \prod \nolimits_{i = 1}^{n} m_{i} (S_{i} )}}{1 - K}$$

7 $$K = \mathop \sum \limits_{{S_{1} \mathop \cap \nolimits \ldots \mathop \cap \nolimits S_{n} = \phi }} \mathop \prod \limits_{i = 1}^{n} m_{i} (S_{i} )$$

where $K$ is the normalization factor or the degree of conflict. Final decision can be made through several ways such as choosing a hypothesis with the maximum value of mass, belief or plausibility. In this paper, we decide to go for the maximum value of mass function. For the sake of simplicity, maximum Bel is chosen to determine selected hypothesis [29–31].

**Fig. 6:**
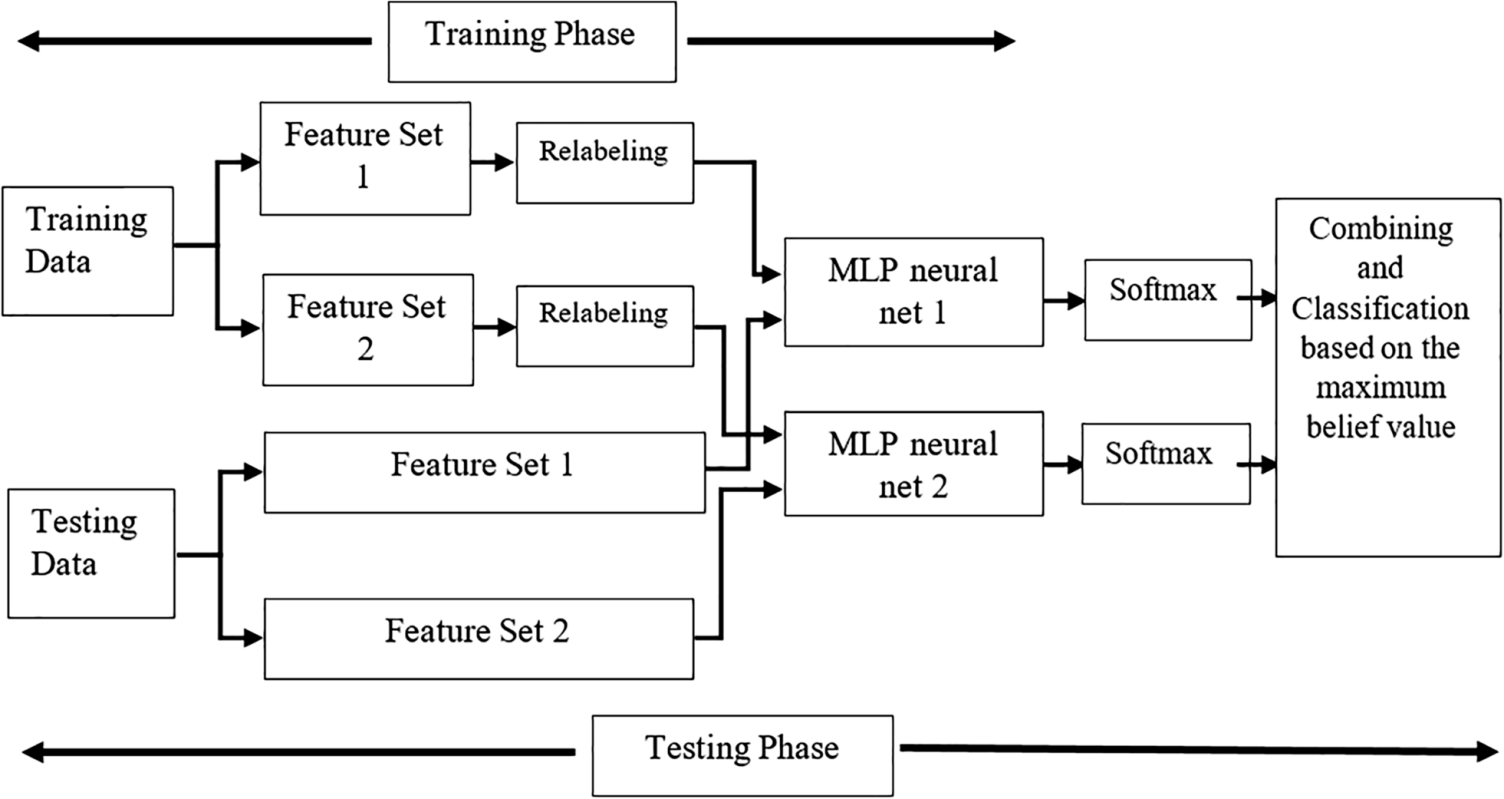
Flowchart of the proposed FBS-based emotion recognition system

To clarify more, it should be noted that in the training phase relabeling should be done in order to put the problem into DST framework. Relabeling is carried out based on what is suggested in [53]. The Euclidian distance between each class prototype and each training sample is calculated. Then a membership function is defined based on the distance which determines the level of ambiguity in the data. Membership values for each training sample is thresholded. A training sample might be assigned to a specific class or a set of classes based on the membership values and the considered threshold. Figure 6 shows the classification procedure in this study.

In testing phase, samples are classified through trained MLPs and the output is normalized using the softmax operator to follow belief function properties. For more information about combining MLPs using DST refer to [55].

In the present paper, two different feature subsets are extracted. Relabeling is carried out for each subset and then two MLPs are trained. In testing phase, MLP outputs are normalized using softmax operator to have belief functions. Based on DST, belief functions are combined and final decision for each test sample is made.

### Evaluation

Classification accuracy, that is the ratio of correctly classified instances to the total number of test samples, as well as confusion and confidence matrices were taken into account to appraise the proposed method. Confusion matrix is a table layout that allows visualization of the classification performance. Each row of the matrix demonstrates the test samples in a predicted class while each column denotes the test instances in an actual class, or the other way around.

#### Confidence matrix

There are several evaluation methods to ensure acceptable and reliable classification results. One of the most widely-used methods is K-fold cross validation where the data set is divided into 10 subsets, with one subset being retained as the test set and the remaining k-1 being used as training data. In most of the literature, K is chosen as 10 according to the size of the data set.

## Results

We present a new method to determine neural activity related to each emotion class which results in EEG emotion-related channel selection. For each BSS method, 32 EEG sources and consequently neural activity maps are reconstructed and then averaged over all samples in each emotional state. Common channels over four emotion classes are considered for the next step. All mentioned features in Table 1 are extracted from the selected EEG channels for all samples. The same features are extracted for each emotion class. These feature have been claimed to be effective in emotion recognition based on the previous studies. The proposed method of feature selection determines features representing and describing arousal and valence values the best. The main idea of this method is to formulate the problem of local feature subset selection as a sequential decision making problem in which we look for a series of good splitting actions. We suggest a sequential decision making process to create feature trees. In other words, the suggested method partitions the sample space into localities and select features for them. The partitions and the corresponding local features are represented using a novel notion of feature tree. As mentioned before, arousal and valence are two major quantities which describe emotions and emotional states. Taking this in mind, we divide the sample space into two main parts and finally we achieve two localities (i.e. arousal and valence) and consequently two subsets of features. Ten most significant features in each subset are selected and finally these features (for train and test samples) are fed into MLPs and DST in order to classify emotions.

Table 2 demonstrates classification methods with respect to different classifiers and algorithms. All implementations are performed using MATLAB (release R2016a) running on Windows 7 Laptop PC with Intel(R) Core (TM) 2 Duo 2.0 GHz processor with 4 GB RAM. As it can be seen, four well-known BSS methods, four most common classifiers and the proposed method are employed and the results are presented in Table 2. For each BSS method and classifier, accuracy and processing time are reported. Besides, statistical analysis using one way ANOVA test is carried out and p-values are represented. Significant differences are in the bold face for each BSS method as well as each classification model. Taking a closer look, we can easily conclude that the proposed Classification method and SOBI are the best combination. Moreover, the proposed features are almost successful in all classification schemes. This suggests that nonlinear features can describe emotions appropriately.

**Table 2:** A comparison among source separation algorithms with respect to different classifiers

	Runica	SOBI	COMBI	JADE	p-value
Index channels	14	16	17	15	–
MLP					
Accuracy (%)	77.16	79.57	76.33	80.28	0.0646
Time (min)	118.46	120.78	116.89	113.45	
KNN					
Accuracy (%)	79.11	81.46	77.16	73.28	0.0894
Time (min)	112.56	110.32	118.96	103.52	
Bayes					
Accuracy (%)	82.57	84.65	78.24	79.67	0.0743
Time (min)	121.32	122.85	119.65	118.45	
SVM					
Accuracy (%)	84.65	86.78	85.96	83.13	0.0531
Time (min)	115.43	112.47	108.75	111.65	
Modified DST					
Accuracy (%)	88.49	*90.54*	86.72	89.32	*0.0417*
Time (min)	122.25	120.82	123.67	126.95	
p-value	0.0631	*0.0301*	*0.0472*	0.0787	

The results suggest that the modified Dempster Shafer method can significantly separate different classes of emotions when second order blind identification (SOBI) algorithm is applied. On the other hand, ranking the channels led to presenting the corresponding channels for each emotion. Having implemented the selecting threshold, the more considerably active channels associated with each emotion were eventually selected, and presented in Table 3.

**Table 3:** A comparison among the values of the selected electrodes in each quarter with respect to source separation algorithms

	Q1	Q2	Q3	Q4	Intersection
Runica	Fp1, Fp2, Fz, F4, F3, F8, Cz, C4, C3, Pz, P3, T4	Pz, P4, P3, F4 O1, T4, F3	T3, T4, C3, T6, P3, T5, P4, F4, O1	P3, T4, F4, Pz, P4, O1, O2, T6, T5, F3	F3, F4, O1, T4
SOBI	Fp1, Fz, F4, F3, F8, Cz, P4, Cz, Pz, P3, O2	Pz, P4, P3, O2, Cz, F3	F3, T4, C3, T6, P3, T5, Cz, O2	P3, Cz Pz, P4, O1, O2, T6, T5, F3	Cz, O2, F3
COMBI	Fp1, Fp2, Fz, F4, O1, F8, Cz, C4, C3, Pz, P3, T4	Pz, P4, P3, O1, T4, F3, FP1	T3, T4, C3, T6, P3, T5, P4, O1, Fp1	P3, Fp1, Pz, P4, O1, O2, T4, T5, F3	O1, Fp1, T4
JADE	F3, Fp2, Fz, F4, F3, F8, Cz, C4, C3, Pz, P3, O1, T4	Pz, P4, P3, O1, T4, F3	T3, T4, C3, T6, P3, T5, F4, F3, O1,	P3, F4, Pz, P4, O1, O2, T4, T5, F3	F3, O1, T4

Afterwards, the intersection between the selected channels was computed. According to the results, the number of selected channels is much lower in other methods indicating that activated regions are approximately constant in each emotion (regardless of the source separations methods).

As Table 3 signifies, temporal areas are prominently more active when experiencing happiness, whereas central and frontal areas play a more significant role in Class 4 emotion, i.e. sadness.

According to Table 2, the modified Dempster Shafer method produces better performance results in comparison with other blind source separation algorithms. Therefore, confusion and confidence matrices are computed to evaluate the errors of the presented method. As shown in Table 4, the desired label value for each class and decided class are defined and at the end, CCR value is reported as 0.9054, which is more appropriate. It should be noted that Q1 to Q4 refer to four different emotion classes according to the arousal–valance plane containing 458, 296, 260 and 266 samples (total = 1280), respectively.

**Table 4:** Confusion and confidence matrices of the proposed method

	Target			
	Q1	Q2	Q3	Q4
Decision				
Q1	407	8	9	10
	88.86%	2.70%	3.46%	3.75%
Q2	23	268	8	3
	5.02%	90.54%	3.07%	1.12%
Q3	17	13	236	5
	3.71%	4.39%	90.76%	1.87%
Q4	11	7	7	248
	2.40%	2.36%	2.69%	93.23%

The upper value in each cell represents the number of samples correctly classified through the proposed method

8 $${\text{CCR}} = \frac{{\mathop \sum \nolimits_{i = 1}^{4} Q_{ii} }}{{\mathop \sum \nolimits_{j = 1}^{4} \mathop \sum \nolimits_{i = 1}^{4} Qji}}$$

**Fig. 7:**
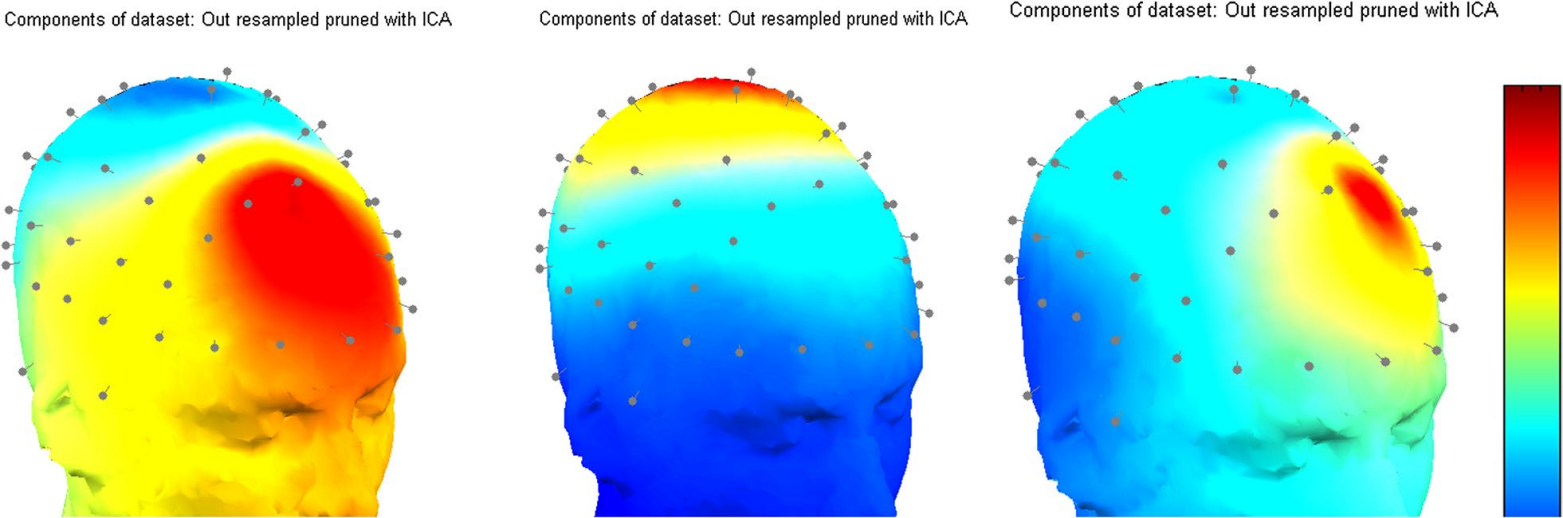
Average activation in brain regions in emotions: **a** Q1, **b** Q2, Q4, **c** Q3

As mentioned earlier, identifying the correlations between different emotions and brain regions has remained a major challenge in the field of emotion recognition. According to the proposed structure, which includes averaging the corresponding values of active regions through various trials, this study introduces average activation within brain regions for each class of emotion. Figure 7 reports average activation in brain regions for 320 trials in various emotions. The most striking results to emerge from the data analysis is that the frontal region is particularly activated when experiencing emotions in Q1 quarter, also, temporal and occipital regions activation evidently correlate with experiencing emotions in Q2 and Q4 quarters, respectively.

## Discussion

As mentioned, detection of brain regions that associate with an emotion is a matter of leading importance in the field of BCI and cognitive sciences. The current study has been able to successfully identify these regions through applying novel methods of feature extraction, selection of emotion-related features, and implementation of Dempster Shafer method as well as upgrading the classic methods. Moreover, this research has made use of blocks containing novel approaches in emotion detection, each of which has the capacity to have improved the results on its own. As one of the novelties, this work uses each of these fully-automated blocks to serve the purpose.

Dempster-Shafer theory is quite well-known in pattern recognition while the classification problem contains uncertainty. In emotion recognition, previous studies such as [54] have employed DST in order to identify emotions through facial expression. It shows that emotion classification is quite subject-oriented and includes imperfect data with uncertain labels. Based on the results of the current study and [54], DST seems to be an effective method of classification in both facial and EEG-based emotion recognition. Since in several samples, individuals did not reflect a specific emotion, DST should be used to decrease the uncertainty.

Some studies such as [56] have tried to classify emotions into four quadrants like what has been done in this work. Emotions are mostly described by arousal and valence which result in arousal-valence plane with four quadrants. In [56] three EEG channels (Fz, Cz and Pz) are claimed to be the most important information sources in emotion recognition. This proves the findings in Table 3 and Fig. 7. Although they have tried to develop a real-time system by means of processing event related potential (ERP), the classification performance is still low.

DEAP dataset has been known as a reliable and rich dataset in emotion recognition. Also, it is noted in numerous studies like [57–60] that visual emotion elicitation has more influential effects. Those mentioned studies, like us, have used DEAP EEG signals. These signals can be considered information sources whereby we can classify emotions. Among these sources EEG has very high spatial and temporal resolution. In addition EEG signals are easily available and price effective.

Most emotion assessment methods consist of three main steps including the biological signal which is processed, extracted features and the classification model. Extracted feature may come from traditional approaches or modern ones which are more related to nonlinear analysis. For example [59–62] employed discrete wavelet transform (DWT) to extract EEG bands and classify emotions while it should be mentioned that DWT cannot exactly and efficiently extract and separate EEG bands since it totally depends on the wavelet kernel [56–59] report that EEG spectral analysis can solve the problem and results in a higher recognition performance while those approaches seem to be still limited and unsuccessful in comparison with the recent methods which apply nonlinear analysis. We can see that both traditional and modern processing approaches have been employed to classify emotions. But common traditional methods which focus on time domain statics, frequency or frequency-scale domain are mostly useful for analyzing linear signals with specific mathematical characteristics such as linear, stationary and Gaussian distributed [63]. However, it is obvious that biological systems such as brain are inherently complex, non-Gaussian, nonlinear, and non- stationary [64]. That is the reason why nonlinear analysis has gained a lot of attention as a novel methodology over the past years. Nonlinear analysis makes it possible to extract more meaningful information and features from the recordings of brain activity [65]. In this study, we focus on EEG nonlinear analysis by extracting features mostly related to signal phase space. Results show that the proposed features are effective.

This research also contributes to the existing literature through organizing the recently proposed approaches.

Identifying active regions for each emotion not only extends our knowledge and ability in the field of BCI, but also comes in particularly useful in diagnosis and treatment applications for mental diseases such as depression, autism etc. Studies in the literature review suggest [19–24] that several emotions at Q1 originate from temporal region, which is near auditory region, this can aid in mental illness treatments. Also, correlations between the active brain regions and emotions in Q3 quarter reveals that, from a psychological perspective, it would be enough to expose the aforementioned regions to electromagnetic waveforms to change the brain mode.

The current study also provides considerable insight into the distribution of activated brain regions associated with different emotional states.

**Fig. 8:**
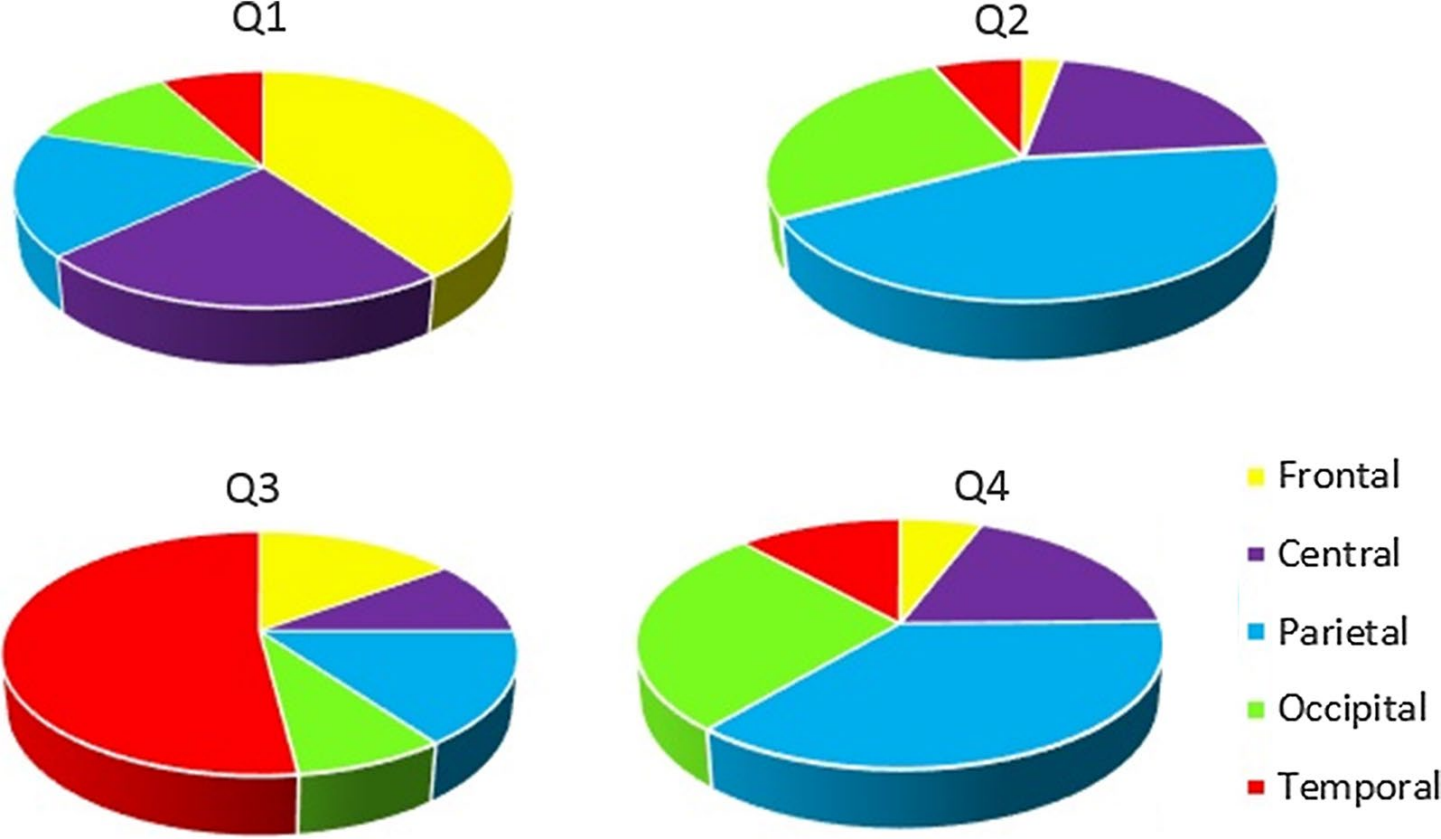
Share of activation of each brain region for each class of emotion

Figure 8 provides a comparison of the share of activation of each brain region while experiencing different classes of emotion. As illustrated, emotions do not originate from a single, specific region but rather from interconnected regions. However, this should not be taken to mean that each region will be equally activated. With that in mind, a strong point of the current study lies in identifying the dominant regions with respect to each class of emotions.

Table 5 enables comparison among available methods in the literature and the proposed approach.

**Table 5:** A comparison of the provided methods in other papers and the proposed method for Emotion Recognition

Authors	Year	Method	Classification accuracy (%)
Fan and Chou [66]	2018	Recurrence quantification analysis, logistic regression	75.7%
Zhong et al. [33]	2017	Spectral and time features, multiple-fusion-layer based ensemble classifier of stacked autoencoder (MESAE)	77.19% (arousal accuracy), 76.17% (valence accuracy)
Atkinson and Campos [22]	2016	Statistical and spectral features, Hjorth parameters, fractal dimension, minimum-Redundancy-Maximum-Relevance, support vector machine	62.39% (valence), 60.72% (arousal)
Xu and Plataniotis [32]	2016	Power spectral density, stacked denoising autoencoders, deep belief network	85.86% (arousal accuracy of SDAE), 84.77% (valence accuracy of SDAE), 88.33% (arousal accuracy of DBN), 88.59% (valence accuracy of DBN)
Jie et al. [67]	2014	Sample entropy, support vector machine	79.11%
Yin et al. [33]	2017	Spectral and time features, multiple-fusion-layer based ensemble classifier of stacked autoencoder	77.19% (arousal accuracy) 76.17% (valence accuracy)
Tripathi et al. [21]	2017	Convolutional neural networks, deep neural network	58.44% (valence, DNN), 55.70% (arousal, DNN), 66.79% (valence, CNN), 57.58% (arousal, CNN)
Alam et al. [29]	2016	Convolutional neural networks	81.17%
Kumar et al. [25]	2016	Bispectrum, least square support vector machine, radial basis function, linear neural network	64.86% (arousal), 61.17% (valence)
Our work	2018	The proposed method	90.54%

Like every single study, our work has some limitations. The proposed method has different steps and it can be problematic while dealing with datasets such as DEAP containing large number of instances and features. The long processing time could be one of the disadvantages. One proposal to resolve this problem is to select effective EEG channels (like what is carried out in this study) in order to consider just the dominant channels and brain regions related to emotions. Active brain regions and EEG channels related to emotions can be determined through other methods such as connectivity analysis which is more complex and time consuming. Although BSS methods have some shortcomings such as initial criteria and assumptions, they are quite simple and fast to implement. In addition, other evaluation functions can be employed for the wrapper step and therefore, we will have faster convergence of the feature selection algorithm. Using a Monte Carlo scheme for searching, the suggested method is likely to be stable with respect to the changes in the feature subset. But it is noteworthy that the proposed method can be unstable for other datasets and evaluation functions.

## Conclusion

The present study has sought to address the long-standing challenge of finding neural correlates between human emotions and the activated brain regions. It has been stressed that all the regions interconnect and none of them is the sole responsible for any specific emotional state. However, some contribute more than others to certain classes of emotion.

The findings presented in this paper can significantly add to the growing body of literature on emotion recognition. Nevertheless, accurate determination of active regions would not conclude here and is still in need of further investigation. One of the methods which seems to be more appropriate among recent studies is the use of two or more modalities. Since each modality shows a different approach from its own aspect, it is expected that combining modalities would produce better results. Future research can explore fusion of EEG and MEG recordings or EEG-fMRI. Since different emotions have different effects on metabolic behavior of blood in capillaries and electrical activity of neurons, it is recommended to assess adding another modality as well as fusion of various modalities.
